# The Health of London

**Published:** 1905-01-21

**Authors:** 


### The Hospital.

A Journal of

The Medical Sciences and Hospital Administration.

Vol. XXXVII.—No. 956. JANUARY 21, 1905.

### THE HEALTH OF LONDON.

The Report on the Public Health of the metropolis, annually presented to the County Council by Sir Shirley F. Murphy, is a document of no small interest and importance, notwithstanding the fact that its issue is of necessity delayed for what seems a somewhat long interval of time since the actual occurrence of the facts to which it relates. The report just published covers the events of the calendar year 1903; and, like its eleven predecessors, touches upon too large a number of interesting questions to be even considered within the limits of a single article. From a general point of view it may, however, be pronounced to be highly satisfactory; for it affords evidence that the London death-rate for the year concerned had declined to 15·2 per 1,000, the lowest on record; that the infant mortality rate had also declined, and was lower during the year than in any of the principal English towns except Bristol; and that the deaths from phthisis were less numerous by 300 than in 1902. Proof is also given that the sanitary authorities of the various districts of which the metropolis is composed are year by year becoming more and more alive to the importance of the duties committed to them, and more and more anxious that these duties should be diligently and carefully performed.

Under the pressure of the daily growing conviction that the best hopes of the future must centre in the young, it is only natural to turn in the first instance to those parts of the report which deal with the hygiene of childhood, and especially with the methods by which schools may be hindered from acting as centres for the diffusion of diseases. The public is indebted to Sir Shirley Murphy for the first announcement of the highly important fact that the prevalence of certain infectious diseases regularly diminishes as a consequence of the school vacations, and as regularly increases as a consequence of the reassembling of the children; and the recognition of this fact has already led to the adoption of precautions by which the continuity of the vicious circle may, perhaps, ultimately be broken. In 1903, as in former years for which similar observations have been made, both the diminution and the subsequent increase were apparent, and were chiefly exhibited in children of the "school age," that is to say, between the ages of three and of thirteen years. The summer holidays of 1903 covered the thirty-first and three subsequent weeks of the year. In the four preceding weeks the notifications of diphtheria within the school age had amounted to 420, in the four weeks of holiday they fell to 287, and in the four weeks after reassembling they rose to 350. Similar facts were observed with regard to scarlet fever, the notifications of the same three periods being respectively 810, 612, and 789. As regards diphtheria, at least, the comparatively recent discovery of the extent to which the bacillus may lurk in the throats of the convalescent, or even in those of persons who do not themselves fall victims to the disease, has seemed to open a way for preventive measures of importance; and the report indicates what has been done in this direction, more especially by Dr. Brown, the medical officer of health for Bermondsey, who has continued the work of inspection of which he gave some account in 1902. During the first nine months of 1903 the "contacts" submitted by him to examination were generally the children of all ages and the mothers of the families in which diphtheria occurred, but under the guidance of experience the method has since been simplified. On the notification of a case of diphtheria, the inspector visits the house and sees that the necessary precautions are carried out. He then requests the parents to send those of their children who attend school to the Town Hall on the second week after the removal of the case, to have their throats examined. If no bacilli are found, the headmaster and parents are informed that the children may return to school; but, if bacilli are found, the children are still excluded until after another examination. The parents are at the same time advised to apply to their medical attendant for an antiseptic gargle, and are asked to bring the children for a second bacteriological examination at the end of another fourteen days. If any of these "contacts" should in the meanwhile become the subjects of sore throats, the parents are advised to call in their medical attendant at once. The energy and sagacity displayed by Dr. Brown have been effectively seconded by the School Board, which has appointed an assistant medical officer, who, when he sees any particular school or class attacked or threatened with an outbreak of diphtheria, visits and examines the suspicious cases bacteriologically, and immediately notifies the medical officer of health if diphtheria bacilli are found. Dr. Brown states that this combined action has so far worked well, and that he hopes to see it adopted all over London and applied to all schools. He has found the parents willing to co-operate with him by bringing their children to the Town Hall for examination, a willingness which must depend, in no small measure, upon his own tact and judgment, and upon the clearness with which the advantages to be expected have been explained to them. A further precaution against the extension of the disease has been rendered possible by arrangements recently made by the Metropolitan Asylums Board, under which every medical officer of health is informed of the discharge from hospital of the patients admitted from his district, so that they may be brought under observation at the time of their return. Dr. McCleary, the medical officer of health for Battersea, reports that the first information of this kind was received on March 15, and that since then it has been the duty of the lady sanitary inspector to visit the homes to which the patients return, and to advise parents as to the precautions which may be advisable in the circumstances. There can be no question but that such action is likely to be widely beneficial.

